# Cerebral vasculitis associated with *Schistosoma mansoni* infection

**DOI:** 10.1186/1471-2334-12-220

**Published:** 2012-09-14

**Authors:** Guillaume Camuset, Valérie Wolff, Christian Marescaux, Ahmed Abou-Bacar, Ermanno Candolfi, Nicolas Lefebvre, Daniel Christmann, Yves Hansmann

**Affiliations:** 1 Unité des Maladies Infectieuses, Centre Hospitalier Universitaire La Réunion, Site Sud, BP 350, 97448, Saint-Pierre, Ile de la Réunion, France; 2 Département de Neurologie, Centre Hospitalier Universitaire, Strasbourg, France; 3 Institut de Parasitologie, Université de Strasbourg, Strasbourg, France; 4 Service des Maladies infectieuses et Tropicales, Centre Hospitalier Universitaire, Strasbourg, France

**Keywords:** Stroke, Cerebral vasculitis, *Schistosoma mansoni*, Corticosteroid, Praziquantel

## Abstract

**Background:**

Cerebral involvement in schistosomiasis is not rare, but it is underdiagnosed because of the lack of clinical suspicion and the frequency of asymptomatic forms. Neurologic complications are generally supported by granuloma formation around ectopic eggs which have migrated to the brain. Moreover, vascular lesions and cerebral arteritis have been well documented in histopathological studies. Nevertheless, cerebral vasculitis in later stages of the *Schistosoma mansoni* infection have not yet been described in living subjects.

**Case presentation:**

A 28-year-old french woman had a stroke linked with cerebral vasculitis, 6 monthes after returning from Burkina-Faso. At the same time, a *S. mansoni* disseminated infection was diagnosed. She suffered from a new stroke after undertaking praziquantel therapy, which lead us to associate the *S. mansoni* infection and cerebral vasculitis.

**Conclusion:**

This is the first report of such association, since cerebral vasculitis has never been described in later stages of the *S. mansoni* infection. Although the causal link between the two pathologies could not be proved, we suggest that *S. mansoni* is able to cause severe vascular damage in cerebral vessels. Schistosomiasis must be investigated in the event of a brain infarct in young people, particularly in patients originating or returning from an endemic area.

## Background

Schistosomiasis is a parasitic disease which remains a public health problem in sub-Saharan Africa and South America. More than 200 million people are affected by it, and the disease continues to spread [1]. *Schistosoma mansoni* infection needs percutaneous penetration of cercariae, the non adult stage of the parasite. After a short period of bloodstream circulation, the parasites mature into mating pairs of male and female worms which preferentially inhabit the inferior mesenteric veins. Then, adult parasites are able to excrete the eggs. Diagnosis could be done recovering *Schistosoma* eggs from faeces or urine in the adult stage but not during the invasive phase.For the latter, only a serologic test can prove the infection.

In Europe, the disease is increasingly encountered because of the increase in international travel and population migration. Cerebral involvement in schistosomiasis is not rare, but it is underdiagnosed because of the lack of clinical suspicion and the frequency of asymptomatic forms [2]. Neurologic complications generally occur in the later stages of schistosomiasis and are supported by granuloma formation around ectopic eggs which have migrated to the brain. The host’s inflammatory reaction probably plays an important role in explaining the great clinical variation of neurologic forms. Deposition of ova in the brain has been reported [3], and adult worms have also been found in the spinal meningeal vein and intracranial venous circulation [4]. The valveless perivertebral Batson plexus seems to be preferential pathway of the eggs and the adult worm to reach central nervous system. Moreover, autopsic series have shown cerebral vascular lesions in 20% of patients with *S. mansoni* infection [5].

In the invasive phase of schistosomiasis, neurologic symptoms can also occur. In this acute stage known as Katayama fever, 2 to 6 weeks after contamination, neurologic involvement is rare. In a large series of US soldiers returning from a Philippines campaign with acute schistosomiasis, 2.3% of 1200 infected soldiers presented neurologic symptoms [6]. In this early stage, hypereosinophilia is pronounced and seems to be linked with cerebral vasculitis [7]. Nevertheless, cerebral vasculitis in later stages of infection without blood hypereosinophilia have not yet been described in living subjects. We report here a case of severe cerebral vasculitis in a 28-year-old woman, associated with a later stage of *S. mansoni* infection, without hypereosinophilia. This is, to our knowledge, the first report of such association in the literature.

## Case presentation

**Figure 1:**
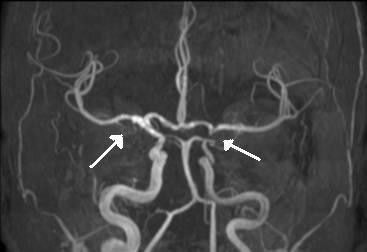
**Bilateral carotid vasculitis.** Angio-MRI showing straight stenosis of the left carotid artery and inflammatory aspect of the right carotid artery (white arrows).

**Figure 2:**
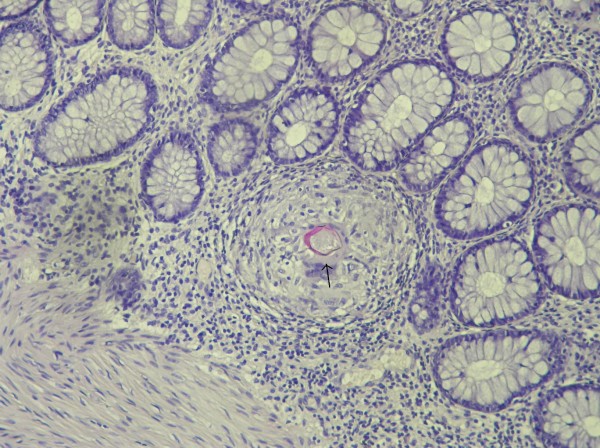
**Rectal mucosa biopsy.** Granuloma around a *Shcistosoma mansoni* egg (black arrow). Magnification X 400.

A 28-year-old woman was admitted to the Neurovascular Unit for transient right hemiplegia associated with language disorders, without fever. The patient complained of unusual headaches since she had returned from Burkina-Faso, 6 months before, where she had worked for a humanitarian organization for one year. She first had a cerebral Magnetic Resonance Imaging (MRI) as soon as she returned to France, which failed to show any disorder. She reported no illness during her mission or before, but had frequently swum in a small lake near where she lived. A cranial MRI was performed at admission, showing multiple vascular hyperintense signals in the left hemisphere. A day after admission, as hemiplegia persisted, an angio-MRI was performed which showed a left junctional infarct, straight stenosis of the left carotid artery and inflammatory aspect of the right carotid artery (Figure 1). No other big trunk was involved on the complete aortic imaging, neither the supra-aortic trunk nor the thoraco-abdominal portion of aorta. Transoesophageal echocardiography did not show any abnormalities. The physical examination showed no hepatomegaly or splenomegaly. The vascular examination found all the pulses. There were no symptoms of arthritis, uveitis or skin manifestation. The white blood cell count was normal, including the eosinophil count (300/mm^3^; normal, < 500/mm^3^). C-reactive protein was normal. By contrast, cerebrospinal fluid showed moderate lymphocytic pleocytosis (28 cell/mm^3^) but without eosinophils. The IgG index was elevated, although the protein concentration remained normal (0.28 gr/l; normal, < 0.50 gr/l). Rheumatoid factor, Cryoglobulinemia, anti-cardiolipid, anti-nuclear and anti-neutrophil cytoplasmic antibodies were negative. Complement titer was elevated. Serologic tests for Human Immunodeficiency Virus, Cytomegalovirus, Epstein-Barr-Virus and viral hepatitis B and C were negative. The serologic test for schistosomiasis was strongly positive in hemagglutination: 1/128 (normal, < 1/8, in-house peparated antigen). Other helminthic serologic tests were negative (trichinellosis, fasciolasis, toxocariasis, anguillulosis). Repeated stool examinations (Kato and Baermann method) failed to detect any *Schistosoma* ova or other helminth ova. To substantiate the diagnosis of disseminated schistosomiasis, a rectal biopsy was performed, confirming the presence of *S. mansoni* granuloma in rectal mucosa (Figure 2).

Treatment with anticoagulant and steroid therapy (prednisone 1 mg/kg/day) was started immediately, with rapid improvement of neurologic symptoms. A few days later, a first-line course of praziquantel was given (40 mg/kg/day for 3 days) and was well tolerated. Ten days after admission, the patient was discharged from hospital with oral anticoagulant therapy and prednisone to be tapered progressively. One month later, a second course of praziquantel was prescribed while she was still taking prednisone at 20 mg/day. The day after she took praziquantel, she complained of a headache and diplopia. The neurologic examination showed right cranial nerve VI paralysis. She was admitted to hospital and a new angio-MRI revealed stenosis of the right carotid siphon. Exacerbation of vasculitis after the praziquantel therapy was suspected and intra-venous methylprednisolone therapy was started (500 mg/day) with resolution of diplopia within 24 h. Oral corticotherapy (1 mg/kg/day) was prolonged for 6 weeks and progressively tapered. The patient did not relapse when prednisone was discontinued. Anticoagulant therapy was maintained for a longer period because of persistent carotid stenosis. A new angio-MRI 6 months later showed minor improvement of both carotid stenoses. The serologic level progressively decreased: 1/32 one year after the first neurologic episode.

## Discussion

Neuroschistosomiasis is an entity which has been thoroughly described in the literature. The spinal form is more frequent than the cerebral form in *S. mansoni* infection, probably because of the large size of *S. mansoni* ova (150 μm long). In fact, the large size of the ova and the lateral spine’s ability to block ova progression along the Batson plexus and the vertebral venous usually stops ascension to the brain [8]. Nevertheless, ova and even adult worms [9] can sometimes reach the brain. Once ova are blocked, they are able to cross the vascular wall and deposit into medullary or cerebral parenchyma. At this time, granuloma formation usually starts around them. Moreover, *S. mansoni* ova can interact with endothelial cells and provoke an inflammatory reaction [10,11] leading to vascular damage, such as necrotizing vasculitis [12]. Thus, the pathophysiology of vasculitis in the latter stages of neuroschistosomiasis (after onset of egg laying) can stem from the ability of eggs to interact with endothelium and provoke an inflammatory reaction in the vascular wall, decreasing the vessel lumen and thrombosing. Host immunity may explain why some people have an asymptomatic form of neuroschistosomiasis [2]. On the other hand, in the invasive stage, the pathophysiology is less clear because the ova cannot be involved in the pathogenesis of vasculitis as they have not yet been laid. Eosinophil-mediated toxicity has been suggested to explain vasculitis and small-vessel thrombosis in acute neuroschistosomiasis [7].

We describe here a case of stroke in a young woman characterized by substantial vascular involvement at the origin of junctional territory infarct. At the same time, diagnosis of schistosomiasis is supported by both the highly positive serologic test and *S. mansoni* ova found in the rectal mucosa biopsy. We cannot exclude a primary angiitis of the central nervous system, but vascular imagery usually shows alternating stenosis and narrowings of blood vessels in this pathology [13], and such findings were not present in our patient. Therefore, differential diagnoses of carotid disease have to be discussed. In this case, we don't have any arguments for auto-immune or Takayasu disease, cancer or lymphoma after two years of follow-up.

A brain biopsy was not performed because of the high risk and insufficient benefits in our opinion, so we are unable to conclude wether there is a causal link between schistosomiasis and carotid vasculitis. Nevertheless, diplopia arose immediately after the second praziquantel treatment which suggests that such a therapy contributed to an exacerbation of vasculitis, which was no longer controlled by the low dose of prednisone. The immediate response to high-dose corticotherapy also argues in favour of the inflammatory hypothesis.

## Conclusion

We describe here a young woman who suffered from a stroke linked with carotid vasculitis. At the same time, a *S. mansoni* disseminated infection have been diagnosed. This is the first report of such association, since carotid vasculitis have never been described in later stages of *S. mansoni* infection. Although the causal link between the two pathologies could not be proved, we suggest that *S. mansoni* is able to cause severe vascular damage in cerebral vessels. This case report points out the potential of severe vascular damage in neuroschistosomiasis, emphasizing the need to include it in the differential diagnosis of cerebral vasculitis and brain infarct in young people, particularly in patients originating or returning from an endemic area. Corticotherapy must always precede praziquantel when neuroschistosomiasis is suspected.

## Consent

Written informed consent was obtained from the patient for publication of this case report and any accompanying images. A copy of written consent is available for review by the series editors of this journal.

## Competing interests

The authors declare that they have no competing interests

## Authors’ contributions

All authors contributed to the conception and content of this case report. GC carried out the clinical assessment. GC, VW, CM, AA, EC, NL, DC and YH drafted and revised the manuscript. GC prepared the figure. All authors read and approved the final manuscript.

## Pre-publication history

The pre-publication history for this paper can be accessed here:

http://www.biomedcentral.com/1471-2334/12/220/prepub
